# Prognostic factors and monomicrobial necrotizing fasciitis: gram-positive versus gram-negative pathogens

**DOI:** 10.1186/1471-2334-11-5

**Published:** 2011-01-05

**Authors:** Ching-Yu Lee, Liang-Tseng Kuo, Kuo-Ti Peng, Wei-Hsiu Hsu, Tsan-Wen Huang, Ying-Chao Chou

**Affiliations:** 1 Division of Sports Medicine, Department of Orthopedic Surgery, Chang Gung Memorial Hospital at Chia Yi, (6 West Section Chia Pu Road), Chia Yi Hsien,(613), Taiwan; 2 Chang Gung University, (259 Wen-Hwa 1st Road, Kwei-Shan), Tao-Yuan, (333), Taiwan; 3 Hyperbaric Oxygen Therapy Center, Department of Orthopedic Surgery, Chang Gung Memorial Hospital at Chia Yi, (6 West Section Chia Pu Road), Chia Yi Hsien,(613), Taiwan; 4 Department of Orthopedic Surgery, Chang Gung Medical Center,(5, Fu-Hsing St, Kwei-Shan), Tao-Yuan,(333), Taiwan

## Abstract

**Background:**

Monomicrobial necrotizing fasciitis is rapidly progressive and life-threatening. This study was undertaken to ascertain whether the clinical presentation and outcome for patients with this disease differ for those infected with a gram-positive as compared to gram-negative pathogen.

**Methods:**

Forty-six patients with monomicrobial necrotizing fasciitis were examined retrospectively from November 2002 to January 2008. All patients received adequate broad-spectrum antibiotic therapy, aggressive resuscitation, prompt radical debridement and adjuvant hyperbaric oxygen therapy. Eleven patients were infected with a gram-positive pathogen (Group 1) and 35 patients with a gram-negative pathogen (Group 2).

**Results:**

Group 2 was characterized by a higher incidence of hemorrhagic bullae and septic shock, higher APACHE II scores at 24 h post-admission, a higher rate of thrombocytopenia, and a higher prevalence of chronic liver dysfunction. Gouty arthritis was more prevalent in Group 1. For non-survivors, the incidences of chronic liver dysfunction, chronic renal failure and thrombocytopenia were higher in comparison with those for survivors. Lower level of serum albumin was also demonstrated in the non-survivors as compared to those in survivors.

**Conclusions:**

Pre-existing chronic liver dysfunction, chronic renal failure, thrombocytopenia and hypoalbuminemia, and post-operative dependence on mechanical ventilation represent poor prognostic factors in monomicrobial necrotizing fasciitis. Patients with gram-negative monobacterial necrotizing fasciitis present with more fulminant sepsis.

## Background

Necrotizing fasciitis is characterized by a rapidly spreading necrosis of the superficial fascia and subcutaneous tissue and is associated with a high mortality despite aggressive surgical treatment and adequate parenteral antibiotic therapy [1]. This disease is generally classified into the following categories: Type 1 (polymicrobial infection), Type 2 (infection with a Group A β-haemolytic Streptococcus or *Staphylococcus aureus*), and Type 3 (infection with a gram-negative bacillus such as Vibrio) [2-4]. The incidence of monomicrobial necrotizing fasciitis has recently increased [5-8]. The soft tissue necrosis that typifies these infections is attributable to the release of endotoxins, exotoxins and proteases that threaten the microcirculation leading to vascular thrombosis and may further serve to promote the extension of complex soft tissue injury [9]. Accurate early diagnosis and surgical intervention combined with administration of appropriate parenteral antibiotics have been the cornerstones of necrotizing fasciitis treatment. In addition, hyperbaric oxygen therapy was suggested to improve the microcirculation such that wound healing migh be enhanced and provide an adjunctive alternative to surgical debridement for treatment of necrotizing fasciitis [10-12].

Prognosis for the patient with necrotizing fasciitis is heavily dependent on initiation of appropriate empiric antibiotic treatment. Therefore, approaches that can assist the physician in the rapid identification of the responsible microbial pathogen are needed. It is currently unclear whether the presentation and/or prognosis for patients with monomicrobial necrotizing fasciitis differ as a function of infection with a gram-positive as compared to a gram-negative pathogen. The purposes of this study were to compare the clinical characteristics of patients suffering from these two classes of monomicrobial necrotizing fasciitis and to evaluate the effects of treatments including parenteral antibiotics, debridement and hyperbaric oxygen therapy on these two classes of infection. It was hoped that such information would serve to predict more accurately the outcome for patients with monomicrobial necrotizing fasciitis as well as to provide a guide for better management of this disease.

## Methods

### Patients

Between November 2002 and January 2008, there were 61 patients diagnosed as necrotizing fasciitis who underwent fasciectomy in conjunction with hyperbaric oxygen therapy in Chang Gung Memorial Hospital at Chia Yi. Among them, there were 46 patients with monomicrobial necrotizing fasciitis were included. There were other 7 patients with polymicrobial necrotizing fasciitis and still other 8 patients with necrotizing fasciitis without any pathogen being isolated from ether blood or soft tissue. Approval for this study was obtained from the Chang Gung Memorial Hospital Institutional Review Board (CGMH-992193B). Necrotizing fasciitis was defined by surgical findings, including the presence of grayish necrotic skin, subcutaneous fat and fascia, no resistance of normally adherent fascia to digital blunt dissection, and a purulent discharge resembling foul-smelling dish water. Histopathological tissue specimens were obtained to confirm the diagnoses [13]. Monomicrobial infection was demonstrated by isolation of single bacteria from soft tissue or blood collected in the Emergency Department (ED) and during surgery.

### Treatments and clinical parameters

The treatment protocol included broad-spectrum antibiotic therapy, aggressive resuscitation, prompt radical debridement, adjuvant hyperbaric oxygen (HBO) therapy, and soft tissue reconstruction. Empiric antibiotic therapy with oxacillin and gentamicin was usually prescribed upon suspicion of necrotizing fasciitis infection. However, when a Vibrio infection was suspected based on a recent history of exposure to sea water or raw sea food, a third-generation cephalosporin and tetracycline were administered instead of oxacillin and gentamycin. Intensive care and aggressive resuscitation, including challenge with fluids and inotropic agents, were given to maintain mean arterial pressure above 65 mm Hg [14]. Emergent surgery with endotracheal tube insertion and general anesthesia was performed for all patients. Criteria for ICU admission used at this hospital were based on recommendations of the American College of Critical Care Medicine and the Society of Critical Care Medicine [15]. Patients with multiple co-morbidities, shock or hemodynamic unstable status (systolic blood pressure < 90 mmHg or 20 mm Hg below the patient's usual pressure or mean arterial pressure < 65 mm Hg) were transferred to ICU for intensive care. Mechanical ventilation support was applied in all patients during operation and continued for patients with postoperative respiratory failure. When hemodynamic status became stable, mechanical ventilation was discontinued. Further surgical debridement was performed every other day if progressive necrotic changes combined with a deteriorating clinical presentation were observed. Adjuvant HBO therapy was initiated after patients' extubation and transfer from the intensive care unit to general ward, and continued once daily for 120 minutes. The treatment protocol of HBO therapy was 10 times initially and adjusted according to patients' response. Soft tissue reconstruction was performed until local infection and soft tissue was relatively stabilized. The reconstruction methods included split thickness skin graft and free vascular myocutaneous flap. The reconstruction methods varied according to the degree of soft tissue defect. All reconstruction surgery was performed by plasty surgeons.

Patients with monomicrobial necrotizing fasciitis were divided into two groups according to the result of gram staining for further analyses: those with infections due to gram-positive cocci (Group 1) and those with infections due to gram-negative bacilli (Group 2). Clinical parameters including age, gender, co-morbidities, presenting signs and symptoms, location of infection, laboratory findings at the time of admission, bacteriological findings, Laboratory Risk Indicator for Necrotizing Fasciitis (LRINEC) score [16], Acute Physiological, Age, and Chronic Health Evaluation (APACHE) II score [17], length of hospital stay, and outcomes such as survival and limb salvage were recorded and compared.

### Statistical analyses

Statistical analyses were performed using the Statistical Package for the Social Sciences for Windows (SPSS, version 12.0). The Fisher's exact test was used for discontinuous variables, and Wilcoxon rank sum test was used for continuous variables. Statistical significance was set at a *p*-value of < 0.05.

## Results

### Clinical characteristics and presentation of patients with monobacterial necrotizing fasciitis

The demographic and clinical characteristics of the forty-six patients with monomicrobial necrotizing fasciitis who were examined retrospectively are presented in Table 1. The median patient age was 63 years (range, 22-85). Thirty-five (76%) patients were male. The lower limbs were more frequently involved (67.4%). Thirty-four (74%) patients were immunocompromised; 18 of these patients had diabetes mellitus, 19 had chronic liver dysfunction, 9 had chronic renal failure (Cr > 1.6 mg/dL) [18] including 2 with end-stage-renal disease, and 4 had a history of malignancy (one case of lung cancer, one of bladder cancer, and 2 of hepatocellular carcinoma). Eleven (23.9%) patients were infected with a gram-positive cocci (Group 1), and 35 (76.1%) were infected with a gram-negative bacillus (Group 2). Of the gram-positive cocci, *Staphylococcus aureus *was the most commonly isolated (54.5%), followed by beta-hemolytic *Streptococcus *(36.3%) and *S. viridans*(9.1%). In six *Staphylococcus aureus*, four were *Methicillin-Sensitive Staphylococcus aureus*(MSSA), and two were *Methicillin-Resistant Staphylococcus aureus*(MRSA). Vibrio species were the most commonly isolated of the gram-negative bacilli (65.7%). Among them, 20 were *Vibrio vulnificus*(60.0%). The followings were *Aeromonas *spp., which were isolated in 8 (22.9%) patients, and one case each (2.9%) of *K. pneumonia*, *Escherichia coli *and *Pseudomonas Stutzeri *was identified. *Prevotella *spp., an anaerobic gram-negative bacterium, was found in one (2.9%) patient.(Table 2)

**Table 1:** Group comparison of characteristics (Table 1)

	Gram positive pathogen (n = 11)	Gram Negative pathogen (n = 35)	p value
Age	59 (34, 72)	64(22, 85)	0.138
Gender(Male/Female)	9/2	26/9	1.000
Involved region			
Upper extremity	5 (45.5)	10 (28.6)	0.462
Lower extremity	6 (54.5)	25 (71.4)	
TiOA(day)	7.18 (2, 14)	2.43 (0.5, 14)*	< 0.001
TiOS(hour)	25.64 (2, 72)	11.74 (1, 72)*	0.046
Immunocompromised(DM, liver dysfunction, Chronic renal failure, Malignancy)	5 (45.5)	29 (82.8)*	0.022
Comorbidity			
DM	5 (45.5)	13 (37.1)	0.728
Gout	5 (45.5)	2 (5.7)*	0.005
Chronic renal failure (> 1.6 mg/dL)	2 (18.2)	7 (20)	1.000
Chronic Liver dysfunction	1 (9.1)	18 (51.4)*	0.016
HBV	0 (0)	11 (31.4)*	0.044
HCV	1 (9.1)	11 (31.4)	0.241
HBV or HCV	1 (9.1)	17 (48.6)*	0.032
Liver cirrhosis	0	8 (22.9)	0.619
ICU stay(patient number)	4 (36.4)	26 (74.3)*	0.032
Post-operative mechanical ventilation (patient number)	4 (36.4)	24 (68.6)	0.080
APACHE II score	12.9 (3, 21)	18.9 (2, 34)*	0.015
ICU stay (day)	2 (0, 7)	4.3 (0, 16)	0.077
Hospital stay (day)	35.7 (15, 70)	35.8 (13, 87)	0.800
Amputation	0	5 (10.9)	0.317
STSG	5 (45.5)	21 (60)	0.494
Free flap transfer	1 (9.1)	7 (20)	0.658
Mortality	1(9.1)	6(17.1)	1.000

Data are presented as median (min, max) or frequency (%)

TiOA: duration of symptoms prior to arrival in the ER

TiOS: time of the first surgical intervention from arrival in the ER

APACHE: Acute Physiological, Age, and Chronic Health Evaluation

Chronic liver dysfunction: liver cirrhosis, viral hepatitis(HBV, or HCV)

STSG: split thickness skin graft

*: The difference is significant (p < 0.05)

**Table 2:** Summary of microbiology

	Gram positive pathogen (n = 11)
Staphylococcus aureus	6(54.5%)
MSSA	4
MRSA	2
Streptococcus sp.	
Alpha-hemolytic	1(9.1%)
*S. viridans*	1
Beta-hemolytic	4(36.3%)
*S. pyogenes*	1
Group B *streptoccus*	3

	**Gram negative pathogen (n = 35)**

**Vibrio spp**.	23(66%)
Vibrio vulnificus	20
Vibrio parahaemolyticus	2
Vibrio cholerae non-O1	1
**Aeromonas spp**.	8(23%)
Aeromonas hydrophila	6
Aeromonas sobria	1
Aeromonas caviae	1
**Kleb.pneumoniae**	1
**E. Coli**	1
**Pseudomonas Stutzeri**	1
**Anaerobes**	
*Prevotella *spp.	1

Data are presented as frequency (%)

MRSA: *Methicillin-Resistant Staphylococcus aureus*

MSSA: *Methicillin-Sensitive Staphylococcus aureus*

No significant differences in the parameters of age, gender, upper versus lower limb involvement and time to first surgical intervention were observed between Groups 1 and 2 (Table 1). The duration of symptoms prior to admission to the ED was longer for patients in Group 1 (*p *< 0.001). Patients with a history of gout were more highly represented in Group 1 (*p *= 0.005) whereas patients with chronic liver dysfunction were more highly represented in Group 2 (*p *= 0.016). In addition, immunocompromised patients were more highly represented in Group 2 (*p *= 0.022), and mean APACHE II scores at 24 h post-admission were higher for Group 2 (12.9 versus 18.9, *p *= 0.015).

All patients presented to the ED with an erythematous, tender, swelling lesion (100% for both groups) but the proportion of patients presenting with shock and hemorrhagic bullae was higher in Group 2 (*p *= 0.009 and *p *= 0.013, respectively; Table 3). The mean WBC count was increased in both groups and the increased percentage of immature leucocytes was also noted in both groups. Thrombocytopenia was more frequently observed in Group 2 (*p *= 0.044). The mean LRINEC score for Group 1 was significantly higher than that for Group 2 (6.2 and 4.5, *p *= 0.025). No significant differences in bacteremia, C-reactive protein, hemoglobin, glucose, sodium, or albumin findings were observed between the two groups. (Table 4)

**Table 3:** Group comparison of signs and symptoms

Symptoms and signs	Gram positive pathogen (n = 11)	Gram negative Pathogen (n = 35)	p-value
Fever (> 38.3°C)	8 (72.7)	16 (45.7)	0.171
Hypothemia (< 35°C)	1 (9.0)	4(11.4)	1
Pain and tenderness	11 (100)	35 (100)	1
Swelling and erythema	11 (100)	35 (100)	1
Hemorrhagic bullae	0 (0)	15 (42.9)*	0.009
Shock(< 90 mmHg)	2 (18.2)	23 (65.7)*	0.013
Inotropic support	2 (18.2)	19 (54.3)*	0.045

Data are presented as frequency (%)

*: The difference is significant (p < 0.05)

**Table 4:** Group comparison of laboratory data

Laboratory data	Gram positive pathogen (n = 11)	Gram Negative pathogen (n = 35)	p-value
**Total WBC**			
Leukocytosis(> = 12,000/ul)	8 (72.7)	23 (65.7)	1.000
Leukopenia(< = 4,000/ul)	0	4 (11.4)	0.559
Leukocytosis or leukopenia	8 (72.7)	27 (77.1)	1.000
**Differential count**			
Band formation	9 (81.8)	33 (94.3)	0.238
Band ≧ 10%	3 (27.3)	16 (45.7)	0.320
neutrophilia(> 7,500/ul)	8 (72.7)	25 (71.4)	1.000
Lymphocytopenia(< 1,000/ul)	2 (18.2)	19 (54.3)*	0.045
**Thrombocytopenia**(< 150,000/ul)	0	12 (34.3)*	0.044
**Bacteremia**	7 (63.6)	17 (48.6)	0.497
**Hemoglobin(g/dL)**	12.4 (10.0, 14.1)	12.6 (7.7, 15.8)	0.690
**C-reactive protein (mg/dL)**	178.0 (50, 373)	114.0 (24.4, 354)	0.302
**Glucose (mg/dL)**	265.4 (121, 842)	164.14(91, 436)	0.065
**Sodium (meq/L)**	132.4 (124, 136)	135.3 (127, 141)*	0.044
**Hypoalbuminemia(< 3 g/dL)**	6 (54.5)	24 (68.6)	0.477
**LRINEC score**	6.18 (4, 10)	4.51 (0, 12)*	0.031

Data are presented as median (min, max) or frequency (%)

Chronic liver dysfunction: liver cirrhosis, viral hepatitis (HBV, or HCV)

LRINEC: Laboratory Risk Indicator for Necrotizing Fasciitis

*: The difference is significant (p < 0.05)

### Characteristics of surviving and non-surviving patients

No significant differences in the parameters of age, gender, infection location, or isolated pathogenic bacterium were observed between surviving and non-surviving patients. (Table 5) However, mortality among patients with chronic liver dysfunction or chronic renal failure was higher (*p *= 0.015 and 0.02, respectively). Mortality was also higher for patients with reduced serum albumin values, thrombocytopenia, and immature leukocyte more than 10%(p = 0.036, and 0.008, and 0.015, respectively). All non-surviving patients required mechanical ventilation and transfer to the ICU after the first surgical intervention (*p *= 0.032) and had longer stays in the ICU (*p *< 0.0002). The mean LRINEC score and its components, including hemoglobin, C-reactive protein, glucose, and sodium values, did not differ between survivors and non-survivors. (Table 6)

**Table 5:** Comparison of characteristics between survivals and deaths

	Survivors(n = 39)	Deaths (n = 7)	p value
**Age**	62 (22, 85)	68 (55, 78)	0.668
**Gender**			
Male	30 (76.9)	6 (85.7)	1.000
Female	9 (23.1)	1 (14.3)	
**Underlying disease**			
Viral hepatitis	13 (33.3)	5 (71.4)	0.093
Liver cirrhosis	4 (10.2)	4 (57.1)*	0.012
Chronic liver dysfunction	13 (33.3)	6 (85.7)*	0.015
Diabetes mellitus	16 (41.0)	2 (28.6)	0.688
Chronic renal failure	5 (12.8)	4 (57.1)*	0.020
**Immunocompromised(DM, liver dysfunction, CRF, malignancy)**	27 (69.2)	7 (100)	0.165
**Pathogens**			
Gram positive pathogen	10 (25.6)	1 (14.3)	0.667
Gram negative pathogen	29 (74.4)	6 (85.7)	
**Involved region**			
Upper extremity	11 (28.2)	4 (57.1)	0.193
Lower extremity	28 (71.8)	3 (42.9)	
**Shock at ER**	19 (48.7)	6 (85.7)	0.106

Data are presented as median (min, max) or frequency (%)

Chronic liver dysfunction: liver cirrhosis, viral hepatitis (HBV, or HCV)

*: The difference is significant (p < 0.05)

**Table 6:** Comparison of laboratory data between survivals and deaths

	Survival n = 39	non-survival n = 7	p-value
**Total WBC**			
Leukocytosis(> 12,000/ul)	27 (69.2)	4 (57.1)	0.667
Leukopenia(< 4,000/ul)	2 (5.1)	2 (28.6)	0.104
Leukocytosis or leukopenia	29 (74.3)	6 (85.7)	0.667
**Differential count**			
Band formation	35 (89.7)	7 (100)	1.000
neutrophilia(> 7,500/ul)	29 (74.3)	4 (57.1)	0.385
lymphocytopenia(< 1,000/ul)	16 (41.0)	5 (71.4)	0.220
**Thrombocytopenia**(< 150,000/ul)	7 (17.9)	5 (71.4)	0.009
**Hemoglobin**(g/dL)	12.6 (7.7, 15.8)	12.4 (10.0, 14.7)	0.866
**C-reactive protein (mg/dL)**	135 (50, 373)	97 (63, 227)	0.915
**Glucose (mg/dL)**	194.9 (90, 842)	152 (105, 264)	0.667
**Sodium (meq/L)**	134.6(124, 141)	134.4 (127, 140)	0.988
**Creatinine > 1.6 mg/dL**	5 (12.8)	4 (57.1)*	0.020
**Hypoalbuminemia(< 3 g/dL)**	22 (56.4)	7 (100)*	0.036
**LRINEC score**	5.1 (0, 12)	3.7 (0, 70	0.217
APACHE II	16.9 (2, 34)	21.3 (17, 27)	0.090
ICU stay (patient numbers)	23 (59.0)	7 (100)	0.078
post-OP ventilator (patient numbers)	21 (53.8)	7 (100)*	0.032
ICU stay (day)	2.5 (0, 16)	10.3 (7, 14)*	< 0.001
Hospital stay(day)	36 (13, 87)	34.6 (14, 79)	0.635

Data are presented as median (min, max) or frequency (%)

APACHE, acute physiological, age, and chronic health evaluation

Chronic liver dysfunction: liver cirrhosis, viral hepatitis(HBV, or HCV)

*: The difference is significant (p < 0.05)

## Discussion

Findings of the present strongly support the concept that patients with monobacterial necrotizing fasciitis due to gram-negative bacilli present with different clinical parameters and predisposing co-morbidities as compared to patients with monobacterial necrotizing fasciitis due to gram-positive cocci. A greater number of patients with chronic liver dysfunction resulting from HBV infection, HCV carrier status or cirrhosis were infected with gram-negative as opposed to gram-positive organisms. By contrast, a larger number of patients with gouty arthritis were infected with gram-positive cocci as opposed to gram-negative bacilli. These findings agree with those of Lee *et al*[8]. who found gram-negative bacillary infections to predominate among 42 cirrhotic patients with monobacterial necrotizing fasciitis. Additionally, Yu *et al *[19]. observed a prevalence of gram-positive cocci infections among 15 gouty patients with monobacterial necrotizing fasciitis. In the present study, differences in characteristics and laboratory findings were observed between patients with necrotizing fasciitis due to gram-positive cocci as opposed to gram-negative bacillary infections. The latter group presented with a shorter duration of symptoms prior to arrival at the ED and more frequently with hemorrhagic bullae, septic shock, and thrombocytopenia. Group 2 patients also had higher APACHE II scores within 24 h of admission, indicating that the severity of disease was greater for this group [17]. Accordingly, cirrhotic patients with gram-negative bacillary necrotizing fasciitis were found to be especially prone to concurrent bacteremia and septic shock [8]. More aggressive resuscitation, intensive care and debridement is therefore recommended when gram-negative bacillary necrotizing fasciitis is suspected based upon a history of exposure, coexisting disease, clinical manifestations, and laboratory findings.

The mortality rate for necrotizing fasciitis is high (cumulative average of 34%), and the limb amputation rate in this disease is reported to be as high as 50% [7,8,20]. In the present study, the overall mortality rate was 15.2% whereas the limb amputation rate was 10.9% under the treatment protocol employed. No significant difference in mortality rate was observed between patients with infections due to gram-positive pathogens and those with infections due to gram-negative pathogens (9.09% versus 17.14%, *p *= 1.000). Antibiotic therapies were chosen based on clinical presentation at the ED: a third-generation cephalosporin plus tetracycline when a gram-negative pathogen such as Vibrio or Aeromonas was suspected, and oxacillin plus gentamicin when a gram-positive pathogen was suspected [21-24]. It is well-established that immediate wide excision of all necrotic soft tissue and appropriate antibiotic therapy are essential for a positive clinical outcome. In the current study, hyperbaric oxygen therapy was included in the treatment protocol to further optimize clinical outcomes. Adjuvant hyperbaric oxygen therapy improves neutrophil function, fibroblast proliferation and collagen secretion, each of which is important in infection control and wound coverage. It was possible mortality and amputation rates might be reduced by adjuvant hyperbaric oxygen therapy [10,11]. For example, Wilkinson *et al *reported that hyperbaric oxygen therapy significantly reduced the incidence of amputation in a retrospective cohort study of 44 subjects with necrotizing fasciitis patients [1]. By contrast Hassan *et al*, who treated 67 comparable subjects with hyperbaric oxygen recently found no significant benefit [25]. The characteristic of present study was monomicrobial necrotizing fasciitis receiving adjunctive hyperbaric oxygen therapy. The present study demonstrated the mortality and amputation rates were 10.9% and 15.2% respectively, in 46 patients. More well-designed, prospective, case controlled studies are warranted to assess the potential benefit of adjuvant hyperbaric oxygen therapy in necrotizing fasciitis.

The present study identified chronic liver dysfunction, chronic renal failure, initial thrombocytopenia, hypoalbuminemia, dependence on post-surgical mechanical ventilation, and a longer ICU stay as risk factors for mortality in monobacterial necrotizing fasciitis. These observations agree well with those of others. Liver cirrhosis and cancer, as well as severe hypoalbuminemia, thrombocytopenia, serum creatinine values exceeding 2 mg/dL, and an increase in the band form of leukocytes were previously reported as risk factors for mortality in necrotizing fasciitis.^[8,26^^] ^Furthermore, post-operative dependence on mechanical ventilation and a longer ICU stay, indicating respiratory failure and sepsis, are reported to serve as factors predictive of amputation and death for such patients [19]. Physicians should therefore be aware of the grave prognosis for patients with necrotizing fasciitis and who present with pre-existing chronic liver dysfunction or chronic renal failure, with thrombocytopenia, or with hypoalbuminemia.

Limitations of the present study should be addressed. First, the number of patients examined was small. Second, the patient cohort was derived from a consecutive series of patients who presented to one hospital over a nine-year period; findings may therefore be influenced by diseases indigenous to a particular region over a given time period. In this regard, chronic liver dysfunction is known to be prevalent in Taiwan currently. Third, patients were placed in groups based on gram-stain findings rather than on identification of the specific pathogen responsible for the infection. Although important initial findings were obtained using this approach, the ultimate goal is to characterize the effects of individual bacterial pathogens on the presentation and outcome for patients with monobacterial necrotizing fasciitis.

In summary, a higher incidence of hemorrhagic bullae and septic shock, higher APACHE II scores, a higher rate of thrombocytopenia, and a higher prevalence of chronic liver dysfunction was observed for patients presenting with monobacterial fasciitis due a gram-negative as compared to a gram-positive pathogen. In contrast, gouty arthritis was found to be more prevalent among subjects with monobacterial fasciitis due to infection by a gram-positive as compared to a gram-negative organism. For non-survivors of monobacterial necrotizing fasciitis, the incidences of chronic liver dysfunction, chronic renal failure and thrombocytopenia were higher, and serum albumin values were lower.

## Conclusion

Pre-existing chronic liver dysfunction, chronic renal failure, thrombocytopenia and hypoalbuminemia, and post-operative dependence on mechanical ventilation represent poor prognostic factors in monomicrobial necrotizing fasciitis. Patients with gram-negative monobacterial necrotizing fasciitis present with more fulminant sepsis. Treatment protocols which include aggressive resuscitation, rapid administration of antibiotics and immediate surgical intervention are recommended for all patients presenting with monomicrobial necrotizing fasciitis.

## Competing interests

The authors declare that they have no competing interests.

## Authors' contributions

CYL participated in the design of the study, collected data, performed the statistical analysis and drafted the manuscript. LTK participated in the design of the study, and drafting the manuscript. KTP participated in the design of the study and assisted in the surgery. WHH conceived the study, carried out surgeries, and coordinated the research groups. TWH participated in the design of the study and revision of the manuscript. YCC participated the design of the study and statistical analysis. All authors read and approved the final manuscript.

## Role of the Funding Source

No external funding source was obtained in this study.

## Pre-publication history

The pre-publication history for this paper can be accessed here:

http://www.biomedcentral.com/1471-2334/11/5/prepub
